# Persistence of Asthmatic Response after Ammonium Persulfate-Induced Occupational Asthma in Mice

**DOI:** 10.1371/journal.pone.0109000

**Published:** 2014-10-10

**Authors:** Marta Ollé-Monge, Xavier Muñoz, Jeroen A. J. Vanoirbeek, Susana Gómez-Ollés, Ferran Morell, María-Jesus Cruz

**Affiliations:** 1 Servicio de Neumología, Hospital Universitario Vall d'Hebron, Barcelona, Spain; 2 CIBER Enfermedades Respiratorias (CibeRes), Barcelona, Spain; 3 Departament de Medicina, Universitat Autònoma de Barcelona, Barcelona, Spain; 4 Centre for Environment and Health, KU Leuven, Leuven, Belgium; 5 Department of Cell Biology, Physiology and Immunology, Universitat Autònoma de Barcelona, Barcelona, Spain; French National Centre for Scientific Research, France

## Abstract

**Introduction:**

Since persulfate salts are an important cause of occupational asthma (OA), we aimed to study the persistence of respiratory symptoms after a single exposure to ammonium persulfate (AP) in AP-sensitized mice.

**Material and Methods:**

BALB/c mice received dermal applications of AP or dimethylsulfoxide (DMSO) on days 1 and 8. On day 15, they received a single nasal instillation of AP or saline. Airway hyperresponsiveness (AHR) was assessed using methacholine provocation, while pulmonary inflammation was evaluated in bronchoalveolar lavage (BAL), and total serum immunoglobulin E (IgE), IgG1 and IgG2a were measured in blood at 1, 4, 8, 24 hours and 4, 8, 15 days after the single exposure to the causal agent. Histological studies of lungs were assessed.

**Results:**

AP-treated mice showed a sustained increase in AHR, lasting up to 4 days after the challenge. There was a significant increase in the percentage of neutrophils 8 hours after the challenge, which persisted for 24 hours in AP-treated mice. The extent of airway inflammation was also seen in the histological analysis of the lungs from challenged mice. Slight increases in total serum IgE 4 days after the challenge were found, while IgG gradually increased further 4 to 15 days after the AP challenge in AP-sensitized mice.

**Conclusions:**

In AP-sensitized mice, an Ig-independent response is induced after AP challenge. AHR appears immediately, but airway neutrophil inflammation appears later. This response decreases in time; at early stages only respiratory and inflammatory responses decrease, but later on immunological response decreases as well.

## Introduction

Occupational asthma (OA) is one of the most common forms of lung-related occupational diseases in Europe, and its annual incidence is increasing. It is estimated that 10% to 25% of all adult onset asthma cases are work-related or caused by occupational exposure [1], [2]. More than 400 agents have been reported to cause asthma in the workplace [3]. These agents can be divided into two groups according to their molecular weight: high-molecular-weight (HMW) or low-molecular-weight (LMW) [4]. Persulfate salts are LMW chemicals widely used in various manufacturing processes [5], especially in bleaching hair products, and are capable of causing immunological sensitization and subsequently allergic diseases such as contact dermatitis and asthma. Persulfate salts are acknowledged as the main cause of OA amongst hairdressing professionals [6]–[10].

However, the mechanisms by which these substances induce sensitization and OA are not yet clear as the processes seem to differ from the typical IgE-mediated allergic response. Previously, our research group demonstrated that AP is able to induce an asthma-like response in a validated mouse model of chemical-induced asthma. In these studies, several features of human OA were induced, such as airway hyperresponsiveness (AHR), neutrophilic inflammation, increased levels of total serum immunoglobulin E (IgE), along with T and B cell proliferation and increased levels of IL-4, IL-10 and IL-13, one day after intranasal instillation of ammonium persulfate (AP) [11], [12].

At present, the measure most commonly implemented to avoid OA-induced symptoms is complete removal from workplace exposure [13]. However, there is insufficient scientific evidence to assert that cessation of exposure improves asthma symptoms [14]. It has been shown that in the case of complete avoidance of exposure, fewer than 1/3 of workers with OA recover from their symptoms [15]–[17]. Reduced exposure has been suggested as a possible alternative to full cessation, with the aim of minimizing the adverse socio-economic effects. However, a recent systematic review reports that reduced exposure seems to be less beneficial than removal of the patient from the workplace [15].

In the case of persulfate salts, it is not known how patients evolve once they avoid exposure to the causal agent. Only one study has described the course of AHR and immunological outcome parameters in patients with OA due to persulfate salts. Despite the persistence of asthma symptoms and AHR in these patients, the study reported an improvement in their condition if exposure was ceased [18].

The aim of the present study was to examine the persistence of the asthmatic response after a specific AP challenge in AP-sensitized mice [11]. AHR, lung inflammation and immune response were evaluated at different time intervals after intranasal instillation of AP in dermally sensitized mice.

## Materials and Methods

### Animals

Male BALB/c mice (∼20 g, 6 weeks old) were obtained from Harlan (Horst; The Netherlands). The mice were housed in filter top cages in a conventional animal house with 12 h dark/light cycles and received slightly acidified water and pelleted food (Teklad 2014, Harlan Laboratories, Indianapolis, IN) *ad libitum*. All experimental procedures were approved by the Ethical Committee for Animal Experiments of Hospital Universitari Vall d'Hebron.

### Mouse model of persulfate salt-induced asthma

On days 1 and 8, all groups of mice received dermal applications of 5% ammonium persulfate (AP, [(NH_4_)_2_S_2_O_8_], Sigma-Aldrich, Steinheim, Germany) or vehicle (dimethylsulfoxide (DMSO), Sigma-Aldrich, Steinheim, Germany) on both ears (20 µl). On day 15, under light anaesthesia with isoflurane (Forane, Abbott Laboratories, Madrid, Spain), they received 40 µl of 1% AP or vehicle (saline, 0.9%NaCl) via intranasal instillation (challenge). The experimental groups were DMSO/SAL and DMSO/AP, identified as control groups, and AP/AP identified as the treatment group: the first abbreviation referring to dermal sensitizations (days 1 and 8) and the second to the agent administered via intranasal instillation (day 15). Each group of mice (controls and treatment) consisted of 4–7 animals for each period of time after intranasal instillation: 1 hour, 4 hours, 8 hours, 24 hours (day 16), 4 days (day 19), 8 days (day 23) and 15 days (day 30). The experiments were repeated twice per group.

### Pulmonary function measurement

#### Airway hyperresponsiveness

After intranasal instillation, reactivity to methacholine was assessed invasively using a forced oscillation technique (FOT) with FlexiVent system (Flexivent, SCIREQ; Montreal, Canada) at each time point (1 hour, 4 hours, 8 hours, 24 hours, 4 days, 8 days and 15 days). Mice were deeply anaesthetised by an intraperitoneal injection of pentobarbital (70 mg/kg) (Nembutal, Abbot Laboratories). The trachea was exposed and tracheotomised, and connected to a ventilator controlled by computer. Airway resistance (R) was measured with a “snapshot” protocol and plotted against methacholine concentration (from 0 to 10 mg/ml) and the Area Under the Curve (AUC) was calculated [19].

### Total Serum Immunoglobulins (IgE, IgG1 and IgG2a)

After the methacholine test was assessed, blood was taken by cardiac puncture and pooled (before BAL). Serum samples were obtained and stored at −80°C for further analyses. The Mouse ELISA kits (Bethyl Laboratories, Inc., Montgomery, USA) were used to measure total serum IgE, IgG1 and IgG2a (diluted samples 1/5, 1/12500 and 1/5000, respectively). Measurements were performed according to the manufacturer's instructions, using biotinylated antimouse IgE, IgG1 and IgG2a detection antibodies and horseradish peroxidise conjugate.

### Bronchoalveolar lavage

After blood sampling, bronchoalveolar lavage (BAL) was performed. The lungs were lavaged three times with 0.7 ml of sterile saline (0,9% NaCl) and the recovered fluid was pooled. Total cells were counted using a haemocytometer and the BAL fluid was centrifuged (1000 g, 10 minutes). The supernatant was frozen (−80°C) until further analyses. For differential cell counts, 250 µl of the resuspended cells (100 000 cells/ml; 1400 g, 6 minutes) were spun (Cytospin 3, Shandon, Thermo Scientific, Cheshire, United Kingdom) onto microscope slides, air-dried and stained [May-Grünwald, 5 min (QCA; Tarragona, Spain) and Giemsa, 15 min (Merck, Darmstadt, Germany)]. Counts for the number of macrophages, eosinophils, neutrophils and lymphocytes were performed in 500 cells from each sample.

Levels of interferon-gamma (IFN-γ) and interleukins-2 (IL-2), IL-4, IL-5, IL-10, IL-13 and IL-17A were measured in the first fraction of undiluted BAL fluid by a mouse cytokine magnetic bead panel according to the manufacturer's instructions (Bio-Plex Pro Mouse Cytokine Group I 7-plex Assay, Bio-Rad Laboratories S.A.; Madrid, Spain). Lower limits of detection were 1.56, 3.41, 6.11, 1.85, 1.26, 4.00, 3.02 pg/mL for IFN-γ, IL-2, IL-4, IL-5, IL-10, IL-13 and IL-17A, respectively.

### Measurement of Th2 related cytokines in homogenized lung tissue

After performing BAL, the left lung was removed and homogenized with 500 µl of BSA/PBS 5%. The homogenate was centrifuged (3000 g, 10 min) and levels of cytokines were measured in the supernatant. The pellet was dried and weighed. Concentrations of IL-2, IL-4, IL-5, IL-13, IL-10, and IL-17A were measured using a Cytometric Bead Array Plex (Bio-Plex Pro Mouse Cytokine Group I 7-plex Assay, Bio-Rad Laboratories S.A.; Madrid, Spain). Measured concentrations were corrected for the lung dry weight. Lower limits of detection were 3.41, 6.11, 1.85, 1.26, 4.00, 3.02 pg/mL for IL-2, IL-4, IL-5, IL-10, IL-13 and IL-17A, respectively.

### Lung pathology

After BAL, lungs were instilled with formaldehyde 3.7–4.0% until all lobes were deemed to be fully inflated by visual inspection. Evaluation of lung injury on slides stained by haematoxylin and eosin (H&E) and Masson's trichrome was performed by an experienced pathologist in a blinded manner. A semi-quantitative scoring system was used to grade the severity and extent of inflammation on haematoxylin-eosin stained sections. We graded the thickness of the infiltrate in the interalveolars septa using as follows: 0 (normal)  =  absence of inflammatory cells; 1 (mild) = 1–2 layers of inflammatory cells; 2 (moderate) = 3–5 layers; 3 (severe) = more than 5 layers.

### Data analysis

All data are presented as mean ± standard error of the mean (SEM) and were analysed using the non-parametric Kruskal-Wallis test and Mann-Whitney U-test (Graphpad Prism 4.01, Graphpad Software Inc, San Diego, USA). A level of p<0.05 (two-tailed) was considered significant.

## Results

### Airway hyperresponsiveness to methacholine

**Figure 1:**
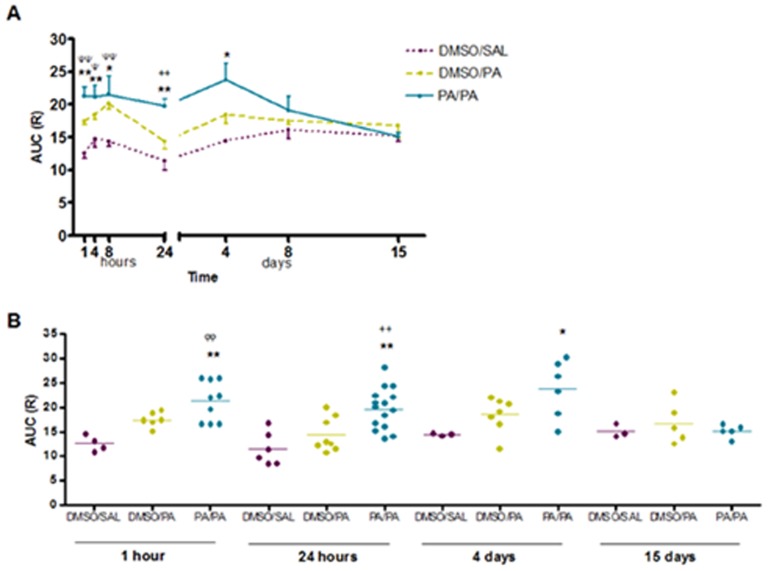
Airway hyperresponsiveness (AHR) to methacholine expressed as resistance (R) was measured 1 hour, 4 hours, 8 hours, 24 hours, 4 days, 8 days and 15 days after intranasal instillation by the forced oscillation technique to increasing concentrations of methacholine. Experimental groups were DMSO/SAL, DMSO/AP and AP/AP. First abbreviation refers to dermal sensitization (day 1 and 8), and the second to the agents administered via intranasal instillation (day 15). **A**) Mean ± SEM of AUC of R against methacholine concentrations (0 to 10 mg/ml) for all periods of time. **B**) Mean individual values of AUC 1 hour, 24 hours, 4 days and 15 days after challenge. *p<0.05, **p<0.01 compared with DMSO/SAL, ++p<0.01 compared with DMSO/AP, Ψp<0.05 and ΨΨp<0.01 when DMSO/SAL is compared with DMSO/AP. No significant differences were found in the other groups studied at different time intervals. AP, ammonium persulfate; AUC, area under the curve; DMSO, dimethylsulfoxide; SAL, saline.

To assess the course of airway hyperresponsiveness (AHR) to methacholine, AUC was calculated for each individual mouse in each experimental group. The airway resistance to methacholine assessed 1 hour after the challenge was significantly increased in AP/AP mice compared with control groups assessed at the same time. This response remained increased until 4 days after inhalation (figure 1). Additionally, significant differences in early AHR (1–8 hours) were found in DMSO/PA groups compared with DMSO/SAL groups. At the later time points (8 and 15 days after the challenge) no significant increases in AHR to methacholine were found.

### Pulmonary inflammation (bronchoalveolar lavage)

**Figure 2:**
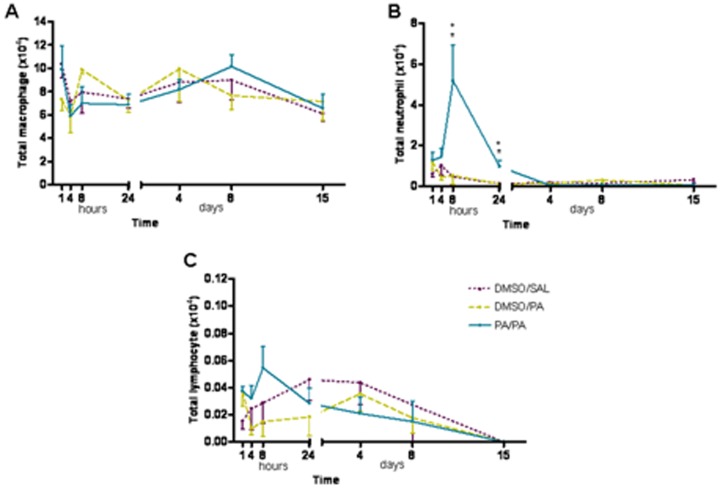
Total number of macrophages (A), neutrophils (B) and lymphocytes (C) in BAL obtained 1, 4, 8 and 24 hours, and 4, 8 and 15 days after AP challenge. Experimental groups are the same as figure 1. Mean ± SEM of total number of neutrophils in BAL. *p<0.05 compared with DMSO/SAL, +p<0.05 compared with DMSO/PA. No significant differences were found in the other groups studied at different time points. AP, ammonium persulfate, BAL, bronchoalveolar lavage, DMSO, dimethylsulfoxide; SAL, saline.

No differences were found in the total cell count in any of groups assessed at any time point. There was a quick response in the total number of neutrophils found 8 hours after the AP challenge in AP-treated mice compared with the control group (DMSO/SAL), which persisted until 24 hours post-inhalation (figure 2B). There were no eosinophils in BAL samples from any of the groups.

**Figure 3:**
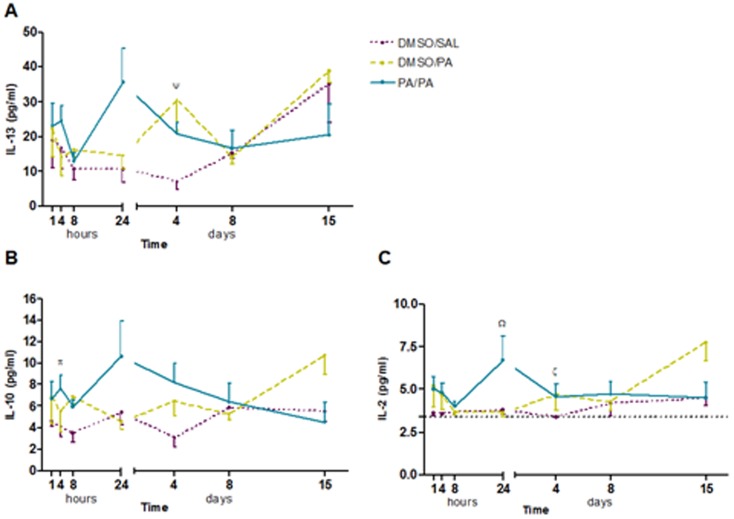
Levels of interleukin (IL)-2, IL-10 and IL-13 in BAL fluid. BAL samples were collected 1, 4, 8 and 24 hours, and 4, 8 and 15 days after AP challenge. Experimental groups are the same as figure 1. **A**) Mean ± SEM of IL-13 concentration. **B**) Mean ± SEM of IL-10 concentration. **C**) Mean ± SEM of IL-2 concentration. Ψ: p = 0.083 compared with DMSO/SAL, π: p = 0.053 compared with DMSO/SAL, Ω: p = 0.055 compared with DMSO/AP, ξ: p = 0.076 compared with DMSO/SAL. No significant differences were found in the other groups studied at different time intervals. AP, ammonium persulfate; BAL, bronchoalveolar lavage; DMSO, dimethylsulfoxide; IL, interleukin; SAL, saline.

Measurement of the cytokines mentioned in BAL fluid revealed increases in IL-10 levels 4 h after AP challenge and increases in IL-2 and IL-13 levels 4 days after AP challenge in the group of AP-sensitized mice, although statistical significance was not reached (p = 0.053, p = 0.076 and p = 0.083, respectively) (figure 3). Neither levels of IL-4, IL-5, nor IL-17A were detected in BAL samples. Cytokine levels were not detectable in tissue homogenate except for IL5, although no significant differences observed between the groups.

### Total serum immunoglobulins (IgE, IgG1 and IgG2a)

**Figure 4:**
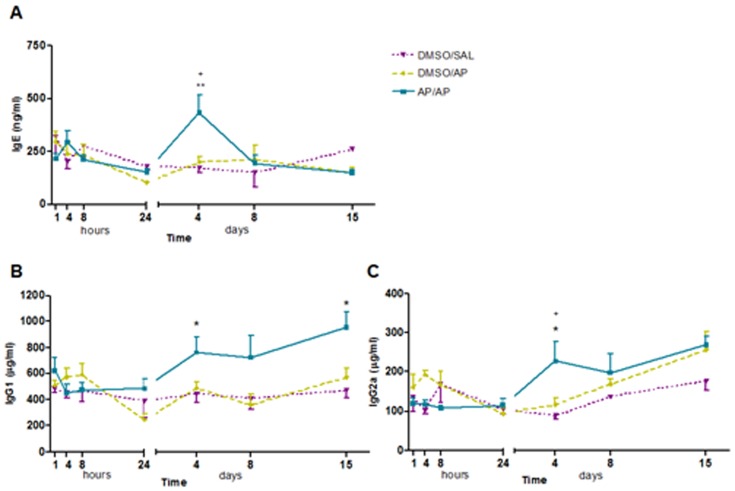
Total serum immunoglobulin (Ig)-E, IgG1 and IgG2a. Blood was collected 1, 4, 8 and 24 hours, and 4, 8 and 15 days after AP challenge. Total serum IgE, IgG1 and IgG2a were measured using a standard ELISA. Experimental groups are as in figure 1. **A**) Mean ± SEM of total serum IgE. **B**) Mean ± SEM of total serum IgG1. **C**) Mean ± SEM of total serum IgG2a. *p<0.05, **p<0.01 compared with DMSO/SAL, +p<0.05 compared with DMSO/PA. No significant differences were found in the other groups studied at different time intervals. AP, ammonium persulfate; DMSO, dimethylsulfoxide; SAL, saline.

Figure 4A shows the levels of total serum IgE at the different time points assessed. AP/AP-treated mice showed significant increases in total serum IgE 4 days after the challenge compared with control groups. However, 8 and 15 days after the challenge no significant differences were found compared with controls, and IgE levels returned to baseline values. Total serum IgG1 increased significantly from 4 to 15 days after the AP challenge (figure 4B), while total serum IgG2a was significantly increased in AP-treated mice 4 days after the challenge (figure 4C).

### Airway histopathology

**Figure 5:**
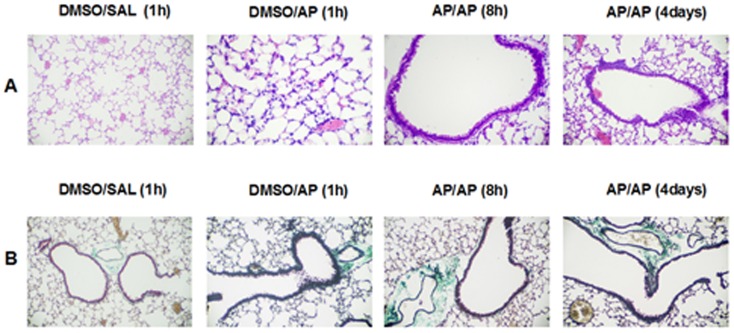
Lung histopathology. Representative images of lung sections are shown at low and high magnification. **A**) Haematoxylin and eosin stained histological lung sections. **B**) Massons's trichrome stained histological lung sections. Experimental groups in this figure are represented with sections from DMSO/SAL, DMSO/AP groups and AP/AP groups assessed 8 hours and 4 days after AP challenge. AP, ammonium persulfate, DMSO, dimethylsulfoxide; SAL, saline.

A blinded histopathological examination of lung tissue sections from the AP/AP mice assessed 8 hours after AP challenge revealed mild to moderate inflammatory cell infiltration and presence of alveolar macrophages compared with control groups. Selectively, at 4 days after the challenge, some moderate peribronchiolar epithilium hyperplasia was observed in the AP/AP group compared with control groups (figure 5A). In this acute single challenge model, no collagen deposition was found, as shown in the lung sections stained with Masson's trichrome (figure 5B). Scoring of stained lung sections illustrates that in AP/AP mice there was an increase in inflammatory cells between 1 and 24 h after challenge (Grade 1, mild), with a maximum at 4 days (Grade 2, moderate). In the DMSO/PA group there was an increase in inflammatory cells 1 hour after challenge (Grade 1, mild) that disappear 4 hours after challenge. No inflammation was observed in the control group.

## Discussion

The present study shows that, in dermally sensitized mice, after exposure to persulfate salts the asthmatic response peaks early after the challenge, and then decreases gradually over time. A reduction in the inflammatory response is observed early, while decreases in airway hyperresponsiveness (AHR) and immunological response occur later on.

Our data show a persistent AHR up to 4 days after intranasal challenge with ammonium persulfate (AP). However, although AHR persisted for several days, a significant increase in pulmonary inflammation was only found within 24 hours after the challenge, with inflammatory cells reaching their peak after 8 hours as shown in both BAL samples and histopathological sections. So there was a clear dissociation in time between AHR and inflammatory response. This type of dissociation between inflammation and AHR has been described in previous studies of patients with asthma. In a study in which the effects of anti-IL-5 were evaluated in patients refractory to high doses of inhaled corticosteroids, a reduction in the number of eosinophils in sputum was observed, but no changes in pulmonary function or AHR were found [20]. Kariyawasam *et al*. [21] studied the involvement of inflammation and airway remodelling in the pathogenesis of AHR. After antigen challenge, in asthmatic patients with a late response, the increased airway inflammation 24 hours after challenge had returned to baseline values after seven days, while increases in AHR and remodelling biomarkers like RBM procollagen III, procollagen I and expression of HSP-47 persisted at this time point. This dissociation in time between AHR and lung inflammation has also been reported in several animal models [22], [23]. The results of these studies suggest that AHR may be the result of independent factors in which inflammation does not have such a relevant direct role. Recently, Hox *et al*. [24], observed AHR without bronchial inflammation after an intranasal challenge with ClO^-^ followed by an ovalbumin challenge in mice. The authors concluded that this AHR is independent of the classic adaptive immunity mechanism. They showed that the induction of AHR may depend on a neuroimmune interaction involving both mast cell activation and the transient receptor potential ankyrin (TRPA)1-dependent stimulation of sensory neurons. This mechanism could explain why the group of non-sensitized mice which received the AP challenge (DMSO/AP) showed early AHR in the present study. In this group, this AHR was not accompanied by an increased number of neutrophils or increased levels of total serum IgE, as happened with the asthmatic groups (AP/AP), demonstrating a possible regulation by a nonspecific irritant mechanism in this case [24]–[26]. It is known that LMW agents elicit an asthmatic response later than HMW agents. Consequently, the early AHR in the previously sensitized asthmatic groups may also be due to this possible irritant effect of the causal agent.

In this model of OA, peak levels of total serum IgE were found 4 days after AP challenge. In some studies in patients with OA due to persulfate salts, the latent period between the exposure and the onset of symptoms and the type of response observed when the challenge test is assessed suggest that OA induced by persulfate salts is mediated by an immunological mechanism [8]–[10]. Positive skin-prick tests for persulfate salts have been reported, suggesting that this immunological mechanism may be mediated by IgE [8], [9], [27], [28]. Nevertheless, the possible role of IgE in persulfate salt-induced OA has not been well established.

Increased levels of IL-2, IL-10 and IL-13 in BAL fluid and IL-5 in tissue homogenate in AP-treated mice were observed after AP exposure, which suggests a mixed Th1-Th2-type immune response in sensitized mice. IL-13 is known for its central role in both IgE production and induction of AHR in allergic humans and mice [29] a finding that is borne out by the results obtained in this study. IL-10 is a cytokine with broad anti-inflammatory properties and has an important role in the regulation of Th2 responses [30]. In an experimental study of allergen exposure in sensitized asthmatic patients, spontaneous increases in the levels of IL-10 produced by *ex vivo* sputum cells were reported [31]. Consequently, the increase observed in the concentration of IL-10 in BAL samples in this study may be due to a compensatory mechanism for the allergic response which occurs after exposure. Finally, IL-2 a typical Th1 cytokine is also linked to the maintenance of Th2 cells, among other activities [32].

A mixed Th1-Th2 response was found not only in BAL cytokines, but also in serum. In this model, both IgE and IgG1 were increased at selected time points. This finding was already reported in another model of chemical-induced asthma [11], [19], [33]. In this study, levels of total serum IgG2a showed the same trend as IgE and remained increased 4 days after AP challenge. While IgE is a typical Th2 response, IgG2a is characteristic of a Th1 immune response. Other animal models using LMW agents to induce asthma have shown similar results in the form of increased levels of serum IgG2a [33], [34]. There is also evidence of this mixed Th1-Th2 immune response in these animals in view of the cytokine profile in cells in the local draining lymph nodes, since the sensitizer compound caused an increase in both Th1-Th2 cytokines [11], [33], [35].

As reported above after the initial peak response, total serum IgE returned to baseline values one week after the challenge. Changes in the levels of total serum IgG1 did not follow the same pattern: total serum IgG1 increased significantly from the fourth day post-challenge and persisted over the two weeks of the experiment. These results are consistent with other studies with animal models of asthma induced by LMW agents, which showed increased total levels of serum IgG (IgG1 and IgG2a) [33], [36], [37].

The role of IgG in response to occupational agents is even more complex. Immunological sensitization to LMW agents is often for life and levels of specific IgG may persist for many years [4]. This IgG persistence was also observed by Vanoirbeek *et al.*, based on animal models of OA due to LMW agents [33]. It has been suggested that IgG1 may be important for monitoring the effect of exposure to LMW agents, and particularly to isocyanates, before the onset of the condition [38], although we did not confirm this possible role in our study. Furthermore, it has been reported that an increase in levels of serum IgG, which matches with the decrease of the AHR and inflammatory response, may have a protective effect in this model of OA [39]. Recent studies with asthmatic patients showed a progressive increase in IgG levels with prolonged exposure to allergens [40], [41].

To our knowledge, this is the first study to assess the persistence of systemic and ventilatory responses in an animal model of OA due to persulfate salts after the end of exposure to the causal agent. Our experiments show that the progressive decrease in the asthmatic response over time observed in mice may mirror that in patients with OA when exposure to the causal agent ceases [14]. However, many of these patients do not completely recover from their asthmatic symptoms [17], supporting the notion that complete removal from the workplace is not more likely to avoid symptoms than continued exposure [14]. In this context, the mouse model described in this study shows evidence that animals exhibit systemic sensitization which makes them susceptible to developing a new asthmatic response when they are re-exposed to the causal agent. This finding has implications for the recurrence of asthma symptoms.
